# Which immunosuppressive drug is preferred in the treatment of toxic epidermal necrolysis during COVID‐19 outbreak?

**DOI:** 10.1002/ccr3.4249

**Published:** 2021-05-15

**Authors:** Saeedeh Farajzadeh, Najmeh Ahramiyanpour

**Affiliations:** ^1^ Department of Dermatology Afzalipoor Hospital Kerman University of Medical Sciences Kerman Iran

**Keywords:** calcineurin inhibitors, COVID‐19, cyclosporine, immunosuppressant, toxic epidermal necrolysis

## Abstract

Cyclosporine is an effective and safe immunosuppressant in the management of Toxic epidermal necrolysis (TEN) during COVID‐19 outbreak for patients that intravenous immunoglobulin (IVIG) is contraindicated or is not affordable.

## Graphical abstract

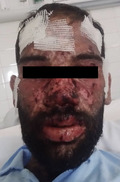

## 1 INTRODUCTION

Toxic epidermal necrolysis (TEN) is a serious drug reaction. Its proper cure would be challenging especially during COVID‐19 outbreak because of a dilemma regarding selecting the immunosuppressive drug. In this case presentation, we report a case of TEN who treat successfully with cyclosporine during COVID‐19 outbreak in a referral COVID‐19 hospital.

Toxic epidermal necrolysis (TEN) is a rare drug reaction associated with high mortality rate.^1^ TEN presents with erythematous and dusky maculopapular rash that progresses to flaccid blisters, epidermal erosion, necrosis, and skin detachment.^1^, ^2^, ^3^ Drug hypersensitivity is the most common cause of TEN. TEN can be associated with infections, systemic diseases, malignancies, and autoimmune conditions.^4^, ^5^, ^6^ While there is no gold standard treatment for TEN, systemic corticosteroid, intravenous immunoglobulin (IVIG), cyclosporine, and tumor necrosis factor (TNF) inhibitors is optional drugs for the management of TEN.^1^, ^2^, ^3^, ^4^

Management of TEN is always challenging especially during COVID‐19 outbreak because use of the immunosuppressive drugs might have both beneficial and harmful effects on COVID‐19 course.^7^ As we know there is not any study to compare the effects of different immunosuppressive drugs such as cyclosporine or prednisolone on TEN patients who are predisposed to COVID‐19. Selecting an immunosuppressive drug in a patient with TEN is challenging and troublesome. Here, we report a case of TEN who treat successfully with cyclosporine during COVID‐19 outbreak in a referral corona hospital.

## 2 CASE SYNOPSIS

A 24‐year‐old man came into the emergency department with a 4 days history of painful generalized dusky rash on his body associated mucosal surface involvement. His past medical history was positive for bipolar mood disorder. He was on carbamazepine and valproate sodium for the last 2 years and lamotrigine during the last 2 weeks.

**FIGURE 1:**
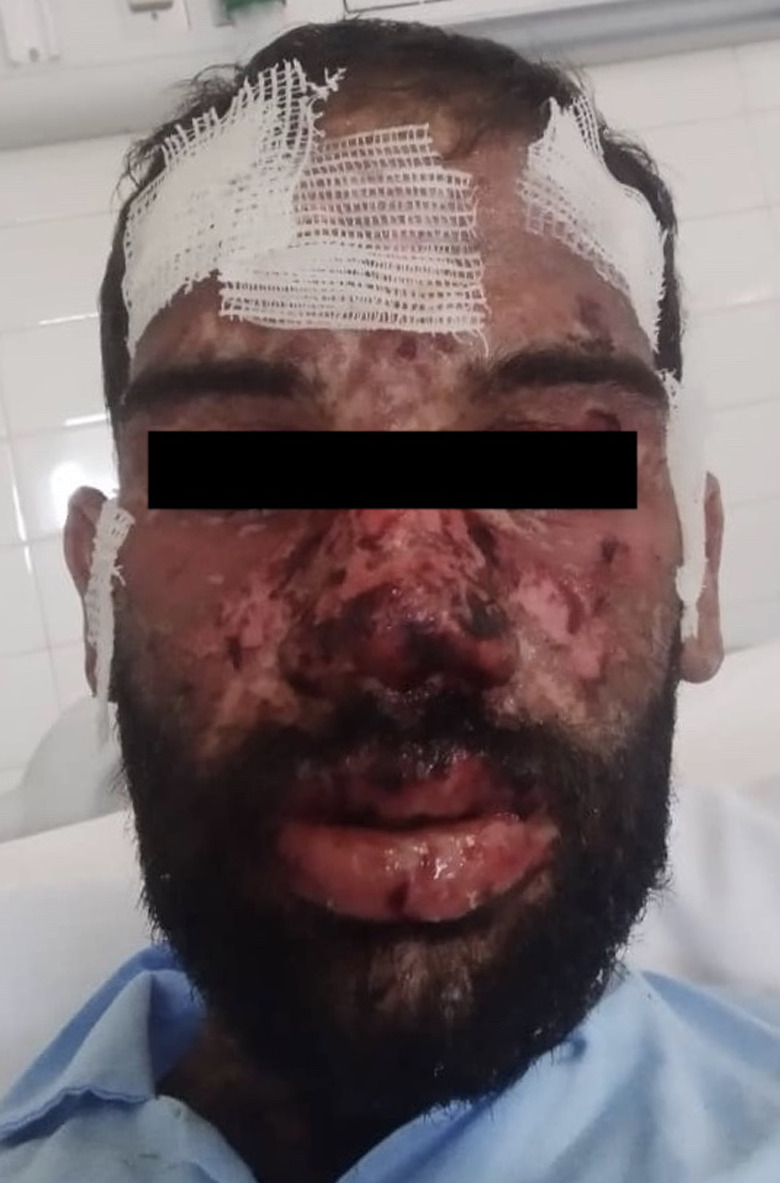
On arrival skin lesions

On arrival to the emergency room, the patient was oriented and his vital signs were stable (Bp: 130/80 mm Hg, pulse rate: 80 beats per minute, temperature: 36.9℃, respiratory rate: 16 per minute, and O_2_ saturation: 99%). Physical examination described more than 50% of body surface area involvement. Skin lesions were as painful, dusky, and purpuric macules and patches of irregular size and shape on the trunk and extremities. Flaccid blisters with serosal fluid and detached epidermal surface on the neck and chest were detected (Figure 1). The Nikolsky sign was positive. Hemorrhagic crust, lips erosion, erythema of buccal mucosa, conjunctivitis, and facial edema are seen He also complained of photophobia and painful dysphagia. His SCORTEN (SCORs of Toxic Epidermal Necrolysis) was 2.

Laboratory tests revealed exclusively a mild leukopenia while other results were negative or in normal ranges: potassium: 4 mmol/L, serum creatinine: 1.1 mg/dL, serum urea: 12 mmol/L, serum bicarbonate: 22 mmol/L, serum glucose: 10 mmol/L, white blood cells: 3.5 μL, hemoglobin: 13.7 g/dL, platelets: 420 000, aspartate transaminase: 24 U/L, alanine aminotransferase: 16 U/L, albumin: 36 g/L.

The patient was admitted with a diagnosis of TEN into the dermatology ward of Afzalipour hospital, of Kerman University of Medical Sciences, Iran that also is a referral COVID‐19 center hospital in Kerman. Lamotrigine was stopped and he was managed with supportive care, wound care, and thrombotic prophylaxis. As corticosteroid is rather controversial during COVID‐19 outbreak and IVIG which is the first choice of treatment was not afforded for the patient, we worked up to start cyclosporine. The blood pressure, electrolytes, and cholesterol were normal. Therefore, we started cyclosporine 4 mg/kg/day.

**FIGURE 2:**
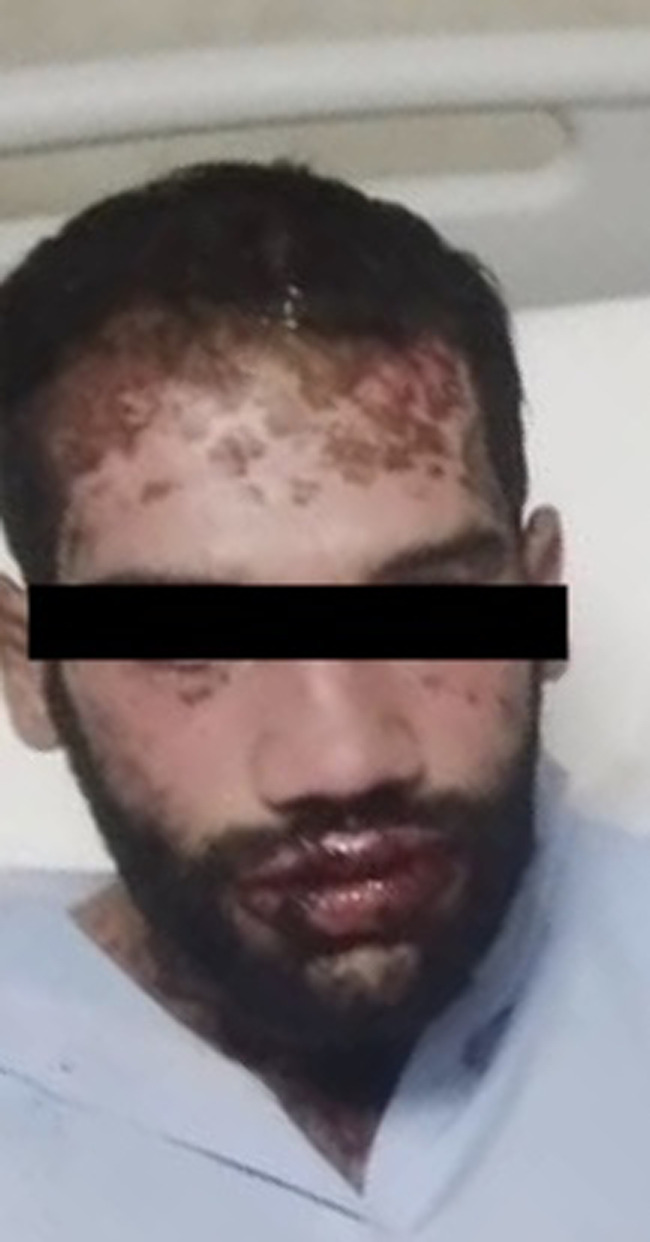
Improved skin lesions at day 4 of admission after starting cyclosporine

Within the first 36 hours after starting cyclosporine, the patient showed a dramatic response to the treatment. No new bullae formation was detected, and there was a reduction in erythema and erosion. The evidence of re‐epithelization was observed on the third day and the Nikolsky sign became negative (Figure 2). Meanwhile, blood pressure and kidneys’ function were normal.

Eventually, he was discharged after 1 week with prescription of the cyclosporin 2 mg/kg/day for another 1 week. He informed respecting the possibility of relapse. One week later, the patient referred to our clinic. Skin and mucosal lesions improved, and the cyclosporine therapy was discontinued.

## 3 DISCUSSION

Although systemic corticosteroid, IVIG, cyclosporine, and TNF inhibitors are optional drugs in managing TEN, it is still controversial about the gold standard treatment for TEN.^1^, ^4^ During COVID‐19 outbreak, the management of TEN has encountered an additive challenge. Because use of the immunosuppressive drug, especially like prednisolone and cyclosporine, is controversial.^8^ It seems IVIG is the best option for the management of TEN, because IVIG is human immunoglobulin and can strengthen the immune system.^9^

An important issue in patients that IVIG is not affordable, as it is the case in our patient, or is contraindicated, in which one of immunosuppressive drugs can be used in the management of TEN during COVID‐19 outbreak. As we know, there is not any study in COVID‐19 outbreak to compare the effects of different immunosuppressive drugs such as cyclosporine and prednisolone on TEN patients.

Use of corticosteroid, which is the routine choice in treating TEN patients, is controversial in COVID‐19 outbreak especially among critical patients.^10^, ^11^ Albeit, it appears that corticosteroids can be effective in severe COVID‐19 cases, its routine use is not supported in the literature.^12^, ^13^, ^14^ On the other hand, due to the increasing mortality of seasonal epidemic of influenza among corticosteroid users, administrating corticosteroids in TEN patients would be of concern.^15^ Therefore, we discarded the use of corticosteroid.

We decided to start cyclosporine, as it can be a good treatment option in COVID‐19 outbreak due to several reasons. First, in Kirchhof et al^16^ cohort study, the relative mortality of TEN patients who were treated with cyclosporine was lower than those with IVIg. Second, cyclosporine might be associated with a rapid re‐epithelialization^17^ and reduce duration of hospitalization,^17^, ^18^ which can reduce the risk of COVID‐19 in critical patients during hospitalization. Third, cyclosporine can inhibit influenza A virus.^19^ forth, in vitro studies showed that cyclosporine can inhibit immunophilin pathway and by this way, it can inhibit replication of coronavirus.^8^, ^19^, ^20^, ^21^, ^22^ Fifth, COVID‐19 mortality is highly linked to the cytokine storm and cyclosporine can be beneficial during the inflammatory phase of COVID‐19.^21^, ^22^ Sixth, Cavagana et al^22^ reported that clinical course of COVID‐19 patients on calcineurin inhibitors (CNIs) is generally mild with a low risk of superinfection. Finally, although cyclosporine is an immunosuppressant agent, infections are not one of its common side effects.^23^

## 4 CONCLUSION

Intravenous immunoglobulin is the best treatment option in the management of TEN during COVID‐19 outbreak. For those IVIG is contraindicated or is not affordable, cyclosporine can be a better choice than other immunosuppressant drugs. As patients on cyclosporine can benefit its antiviral effects and have a shorter hospital admission stay.

## CONFLICT OF INTEREST

The authors declare no conflicts of interest.

## AUTHOR CONTRIBUTION

SF: involved in follow‐up and visit, data collection, and manuscript review. NA: involved in follow‐up and visit, data collection, and manuscript drafting.

## ETHICAL APPROVAL

The subject signed the written informed consent.

## Data Availability

Data are available upon request to the corresponding author, Najmeh Ahramiyanpour MD
